# Neonatal Jaundice Diagnosis Using a Smartphone Camera Based on Eye, Skin, and Fused Features with Transfer Learning

**DOI:** 10.3390/s21217038

**Published:** 2021-10-23

**Authors:** Alhanoof Althnian, Nada Almanea, Nourah Aloboud

**Affiliations:** 1 Information Technology Department, College of Computer and Information Sciences, King Saud University, Riyadh 11451, Saudi Arabia; 437203655@student.ksu.edu.sa; 2 Decision Support Center, King Abdulaziz City for Science and Technology, Riyadh 12354, Saudi Arabia; naloboud@kacst.edu.sa

**Keywords:** jaundice, healthcare, smartphone sensor, diagnosis, machine learning, deep learning, transfer learning, CNN, SVM, MLP

## Abstract

Neonatal jaundice is a common condition worldwide. Failure of timely diagnosis and treatment can lead to death or brain injury. Current diagnostic approaches include a painful and time-consuming invasive blood test and non-invasive tests using costly transcutaneous bilirubinometers. Since periodic monitoring is crucial, multiple efforts have been made to develop non-invasive diagnostic tools using a smartphone camera. However, existing works rely either on skin or eye images using statistical or traditional machine learning methods. In this paper, we adopt a deep transfer learning approach based on eye, skin, and fused images. We also trained well-known traditional machine learning models, including multi-layer perceptron (MLP), support vector machine (SVM), decision tree (DT), and random forest (RF), and compared their performance with that of the transfer learning model. We collected our dataset using a smartphone camera. Moreover, unlike most of the existing contributions, we report accuracy, precision, recall, f-score, and area under the curve (AUC) for all the experiments and analyzed their significance statistically. Our results indicate that the transfer learning model performed the best with skin images, while traditional models achieved the best performance with eyes and fused features. Further, we found that the transfer learning model with skin features performed comparably to the MLP model with eye features.

## 1. Introduction

The fast advancement in information technologies has a tremendous effect on healthcare. Electronic Health (eHealth) is a relatively recent interdisciplinary research area that applies information technologies to improve healthcare processes and services. The term first appeared in 2000 and was since then commonly used [1]. One of the critical areas in healthcare where eHealth has been successfully applied is diagnosis, where the symptoms are examined by a doctor to identify the illness or any other health problems. In fact, artificial intelligence, specifically machine learning and deep learning, has contributed to tackling multiple challenges in the diagnosis of different diseases. Since their emergence, a wide range of research has been carried out with breakthrough results [2,3,4,5]. When symptoms are visible, images of the infected area are collected, and computer vision and image processing algorithms and techniques are applied to extract features that are fed to the diagnostic models.

Neonatal jaundice is a condition that often causes neonates to have yellow skin and eyes due to excess bilirubin. This is caused by hemoglobin breakdown, which is excreted into the liver’s bile. The condition makes the neonate sleep more than expected and have difficulties in breastfeeding, which affects the overall health [6]. Jaundice is usually diagnosed in hospitals by pulling a blood sample to detect the level of bilirubin in blood. Experienced doctors may be able to detect neonatal jaundice by the naked eye but have to perform a blood test to confirm their doubts. Since a high bilirubin level results in yellowish coloring of the skin and eyes, analysis of images of such areas can potentially be used to diagnose neonatal jaundice. Therefore, many efforts have been devoted to automating the diagnosis of neonatal jaundice using different computer vision, image processing, and machine learning techniques. Existing works have used images of two different parts of the neonate’s body, namely the neonate’s skin and eye. For the former, researchers have used images of a neonate’s face [7], forehead [8,9,10,11], sternum [9,10,12,13], abdomen [13,14], or multiple body parts such as sole, palm, forehead, and arm [15], and face, arms, feet, and middle body [16]. For the latter, researchers have used an eye’s sclera for diagnosis [17,18,19,20,21,22]. Further, the study in [23] has focused on stool samples.

Medical practitioners have long relied on the naked eye to diagnose neonatal jaundice. Currently, the diagnosis is usually performed in hospitals and private clinics by taking a blood sample and measuring total serum bilirubin (TSB). However, non-invasive procedures that measure transcutaneous bilirubin (TcB) from the skin have emerged recently, such as BiliCheck [24,25,26], JM-102 Minolta [27]. Diagnosis of jaundice can be challenging for doctors, hence the work in [28] proposed a decision support system for unexperienced practitioners, while the work in [29] provided a guideline to prevent hyperbilirubinemia. Some efforts focused on comparing the accuracy of different diagnostic approaches. For instance, authors of [30] compared a doctor’s naked eye diagnosis and medical devices, and concluded that the former approach is not reliable. This viewpoint was also supported by the work in [31]. However, the authors of [32] found that the clinical assessment of neonatal jaundice by the naked eye of an experienced clinician is still a reliable diagnostic approach.

There are some available non-invasive diagnostic tools, also known as transcutaneous bilirubinometers (TcB), such as BiliCheck [24,25,26], Minolta JM-102 [27]. These tools are based on skin color detection and are only available for medical practitioners in hospitals and not to the public. Moreover, there are a few efforts to develop mobile applications for jaundice diagnosis based on skin features, such as BiliCam [9,12] and BiliScan [13], or eye features, such as Biliscreen [18] and BiliCapture [20]. Authors of [33] presented issues and challenges faced by non-invasive methods in the detection of neonatal jaundice, such as skin type, age, and melanin.

Although a wide range of research has been carried out on non-invasive neonatal jaundice diagnosis, no single study exists which uses fusion of both eyes and skin features. The motivation behind this is to simulate real-life naked-eye diagnosis. It is observed that health practitioners do not rely solely on either eye or skin during naked-eye jaundice diagnosis. Instead, they check both areas simultaneously for further assurance. Moreover, previous studies in this domain have not applied deep learning and transfer learning models, despite their success in the diagnosis of different diseases [2,3,4,5]. In this work, we seek to fill this gap. We investigated the effectiveness of transfer learning in diagnosing neonatal jaundice using different types of features, including skin, eye, and fusion of skin and eye. Further, we compared the performance of transfer learning with multiple machine learning models, including multi-layer perceptron (MLP), support vector machine (SVM), decision tree (DT), and random forest (RF), when trained on the previously mentioned features. Unlike most of the existing contributions, the performance of the models was reported with respect to accuracy, precision, recall, f-score, and area under the curve (AUC). We compared the performance of the models and analyzed their significance statistically. The dataset of healthy and jaundiced neonates was collected from King Khalid University Hospital (KKUH) in Riyadh, Saudi Arabia using the built-in camera in a Samsung S7 smartphone.

The rest of this paper is organized as follows: Section 2 presents the related works. The material and methods are presented in Section 3. In Section 4, we present the results and discussion. Finally, Section 5 concludes the work.

## 2. Related Works

Neonatal jaundice is one of the most common conditions in neonates. It is caused by excessive bilirubin in the blood, which results in yellow pigmentation in the skin, eye, or pale stools of the neonate [6]. A wide range of efforts exist in the literature that analyze images of neonates’ eyes, skin, or pale stool to recognize jaundice [7,8,9,10,11,12,13,14,15,16,17,18,19,20,21,22,23]. Further, there exist a few efforts that do so using numerical data, such as [34,35,36,37]. In the text below, we highlight the main efforts made in the field with a focus on skin and eye images as a source of data. Further, we summarize the results of the most relevant works in Table 1.

For skin-based diagnosis, related efforts have used different parts of a neonate’s body such as face [7], forehead [8,9,10,11,15], sternum [9,10,12,13], abdomen [13,14], or multiple body parts such as sole, palm, and arm [15] and face, arms, feet, and middle body [16]. The authors adopted varying methods for feature extraction such as mean, standard deviation, skewness, kurtosis, energy, and entropy [7], YCbCr and lab color spaces [9,12,14], RGB [11,12,14,15,16], hue and saturation values [11,13], and diffuse reflection spectra features [15]. Different machine learning models have been used for jaundice diagnosis such as K Nearest Neighbor (KNN) [7,14], SVR/SVM [14,15], regression [11,13,16], and an ensemble of multiple classifiers, including (KNN), least angle regression (LARS), LARS-Lasso elastic net, support vector regression (SVR), and random forest (RF) [9,12].

A few works used images of serum bilirubin coloration on detection strips. For instance, Saini et al. [10] used images of neonates’ forehead and sternum skin to detect neonatal jaundice by matching them with images of serum bilirubin coloration on detection strips. Singla et al. [38] has also used bilirubin strip images to examine the effectiveness of homomorphic filtering on jaundice detection. In their work, the authors applied homomorphic filtering on the images’ computed blue color intensities. Correlation was computed between actual and predicted bilirubin levels.

The presence of neonatal jaundice may be detected by the yellowing of the eyes’ sclera due to the accumulation of bilirubin in the body and the insufficiency of the liver to get it out of the body. Multiple efforts exist in the literature for detection of adults’ jaundice using images of eyes’ sclera and a box to control eye exposure to light. For instance, Laddi et al. [21] used a 3CDD camera and a light source covered by aphotic housing made of acrylic paper to capture eye images which were then fed to CIE Lab color model. The work in [18] used an iPhone SE to capture the images with two accessories, a head-worn box, and paper glasses with colored squares for calibration. Their results showed that the box achieved better results. For neonatal jaundice, the work in [17] used a Nikon camera to capture eye images, where RGB features were extracted and fed to a linear regression model to predict TSB levels. Rizvi et al. [20] used the Diazo method with dichloroaniline (DCA) to estimate bilirubin level using neonates’ eyes images. The authors in [19,22] captured two versions for each image, namely with flash and no-flash images. In the latter work, the images are used to apply the ambient subtraction technique, which proved to achieve promising results. In this technique, the RGB values from the two versions of images are subtracted in order to estimate the raw values without ambient illumination. A few studies, such as [17], compared skin and eye images in diagnosing jaundice using linear regression. Their results showed that the latter can achieve better results.

Several methods have been adopted in the literature for data collection. A smartphone camera has been used successfully to capture images of jaundiced and healthy neonates, such as in [8,9,11,12,15,16,18,20,22,38,39]. In contrast, the work in [8] tested several methods, including a direct camera method, a yellowish-green gelatin filter method, and a dermatoscope method, to determine whether a smartphone camera can be used as a screening tool for jaundice. The authors concluded that only the latter method is effective for jaundice detection. Further, the studies in [9,11,12,13,14] used a calibration card for the purpose of color balancing, while the studies in [7,38] did not. In [39], the authors proposed a novel white balancing method with a dynamic threshold for adjusting different color temperatures without the use of a calibration card. The works in [10,38] collected serum bilirubin coloration on stripes. The authors of the work in [18] collected images of the eyes using two different methods, namely closed box and colored glasses, while in [21], they collected the data using only closed box to capture eye images.

As previously mentioned, there are some existing medical devices that measure transcutaneous bilirubin from the skin, such as BiliCheck [24,25,26], Minolta JM-102 [27], and also some efforts to develop smartphone applications based on skin images, such as BiliCam [9,12] and BiliScan [13], and eyes images, such as Biliscreen [18], BiliCapture [20], and neoSCB [22]. A considerable amount of literature has been published on how the performance of these non-invasive bilirubin detection tools compares [24,30,40,41,42]. For example, the work in [30] presented a comparison between JM103, BiliCheck, and BiliCam. The results showed that BiliCam can detect the bilirubin level with high sensitivity and in less time. The study in [24] compared BiliCheck and the Minolta bilirubin meter. The results showed that the correlations between TSB and TCB measurements of the two devices were high. In contrast, the study in [40] compared BiliCheck and Minolta JM-102. The results showed that the accuracy of the former was not affected by the color of the skin, while the other jaundice meter was affected. Similarly, the work in [41] compared Minolta JM-102 and BiliCheck. Their results showed that Minolta JM-102 performed the best on the sternum, while BiliCheck performed better on the forehead than the sternum.

Much of the current literature on neonatal jaundice diagnosis pays particular attention to non-invasive tools based on either eye [17,18,19,20,21,22] or skin [7,8,9,10,12,13,14,15,38] images. A few efforts exist that compare between the two sources of data, such as [17]. However, no attention has been paid to the combination of skin and eye features to diagnose jaundice. Further, very little is known about the application of deep transfer learning models in this domain. This work seeks to fill this gap by examining the efficacy of both traditional machine learning models and transfer learning using skin, eye, and fused features.

## 3. Material and Methods

**Figure 1:**
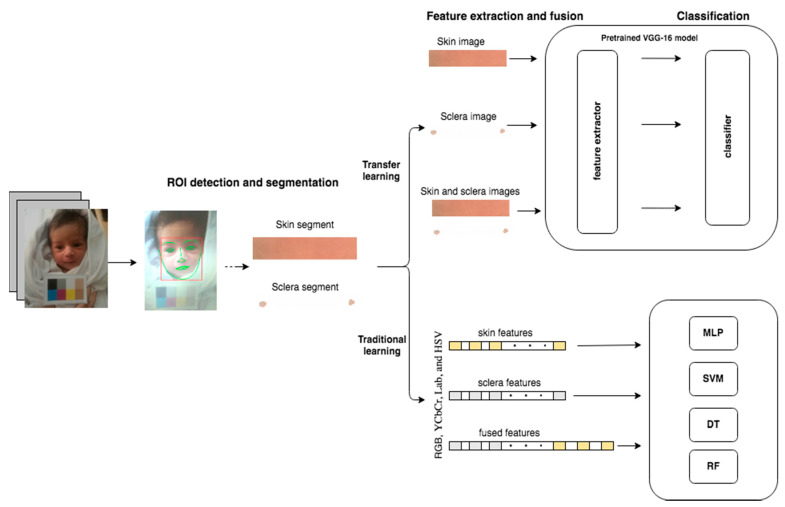
Illustration of the method. Forehead skin and eyes’ sclera regions are detected and segmented from the preprocessed images. The segmented skin, sclera, or skin and sclera images are then passed to (1) transfer learning using a pretrained VGG-16 model for feature extraction and classification and (2) traditional learning to extract RGB, YCbCr, Lab color space, and HSV color space features and then to multi-layer perceptron (MLP), support vector machine (SVM), Decision tree (DT), and random forest (RF) for classification.

An illustration of our method is shown in Figure 1. In the subsections below, we explain each step in further detail.

### 3.1. Dataset

The study has been approved by King Saud University institutional review board (IRB) research project No. E-19-3871. Parents of all study neonates gave informed written consent to participate in the study. Following [20], our inclusion criteria included gestation age between 35 and 42 weeks, neonate’s age between 0 and 5 days, neonate’s weight ranged from 2.00 to 4.35 kg, and lastly, the neonate had to be completely in a quiet state. Exclusion criteria, similar to [13,20], included neonates already in the neonatal intensive care unit, those treated by phototherapy, gestational age less than 35 weeks, or weight less than 2 kg. A total of a hundred neonates in King Khalid University Hospital (KKUH) in Riyadh, Saudi Arabia were accepted to the study between May 2019 and September 2019.

**Figure 2:**
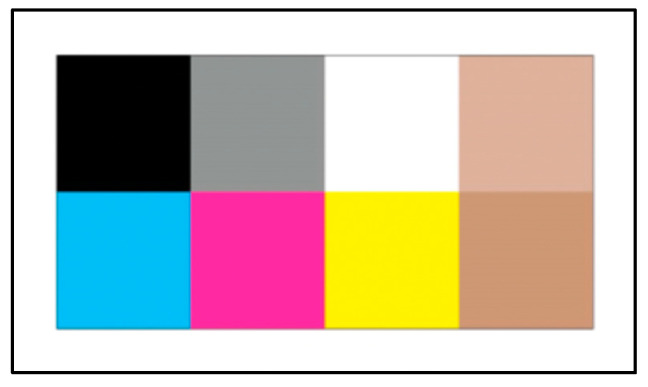
Color calibration card.

Our procedure for collecting the dataset was as follows: We used a Samsung Galaxy S7 mobile phone’s built-in camera to collect the data. We made sure that the neonate was awake in order to capture his/her eyes’ sclera. We placed a calibration card [9] (see Figure 2) on the neonate’s chest. Then, we took a picture or recorded a video of the neonate’s full face including the calibration card under unconstrained conditions of illumination and background. In order to establish a ground truth for the neonates’ jaundice condition, the neonates’ TCB level was measured and recorded by an accompanying nurse using the JM-103 jaundice meter device [27]. After collecting the dataset, a pediatrician at KKUH used the TCB measurements to label neonates as either healthy or jaundiced. A neonate with a TCB level of 204 or above was considered jaundiced, and healthy otherwise.

The collected dataset consisted of 62 male and 38 female neonates, where 67% of them were healthy and 33% were jaundiced. The average neonates’ gestation age was 38 weeks, and the average age was 1 day. The average TCB level was 231 Mmol/M. During feature extraction, we had to eliminate 32 images, since the face recognition algorithm could not recognize the neonates’ faces due to presence of the mother’s hand or pacifier in the image. This left the dataset with 68 samples. Table 2 highlights the dataset characteristics.

### 3.2. Preprocessing

In order to overcome varying lighting conditions in the collected images, similarly to [9,12,13,14,17,18], color balancing [43] was applied to all images using the calibration card. First, the location of the card was detected by using a mask on the phone’s screen to align the card with the mask. Then, the white color patch on the card was identified by counting the number of steps. Next, color balancing (also known as white balancing) was applied by using the observed RGB values of the white color patch to adjust the RGB values of the image using Equation (1) below:

(1) $$\left[\begin{array}{c} R\\ G\\ B \end{array}\right]\;=\left[\begin{array}{ccc} 255/R_{w}^{\prime } & 0 & 0\\ 0 & 255/G_{w}^{\prime } & 0\\ 0 & 0 & 255/B_{w}^{\prime } \end{array}\right]\left[\begin{array}{c} R^{\prime }\\ G^{\prime }\\ B^{\prime } \end{array}\right]$$

where *R*, *G*, and *B* are the color balanced red, green, and blue components of a pixel in the image, and *R*′, *G*′, and *B*′ are the red, green, and blue components of the image before color balancing, respectively, and $R_{w}^{\prime }$, $G_{w}^{\prime }$, $B_{w}^{\prime }$ are the average colors of the white patch on the color calibration card [43].

### 3.3. ROI Detection and Segmentation

The first step to extract features from neonates’ eyes and forehead skin was to detect and segment the regions of interest, i.e., eyes’ sclera and forehead skin. For this, Dlib OpenCV Face Landmark Detection [44], which is a pretrained detector for face landmarks, was used.

**Figure 3:**
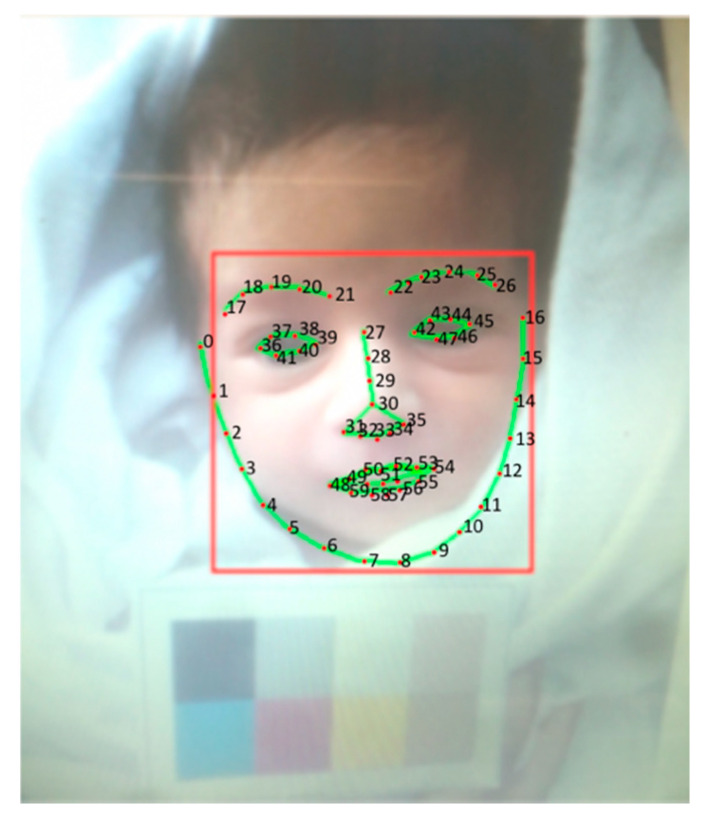
Face landmarks detection.

The detector can define face features by predicting the position of 68 points in the face to determine face landmarks such as eyes, nose, mouth, forehead, eyebrows (see Figure 3). For forehead segmentation, we focused on the area twenty pixels above the points in the range [18, 25] to avoid the eyebrows, and we counted 120 pixels above to be the height of the area [45]. To determine the sclera of left eye, we focused on points in the range [42, 47], while for the right eye, we used the area in the range [36, 41].

### 3.4. Transfer Learning

**Figure 4:**
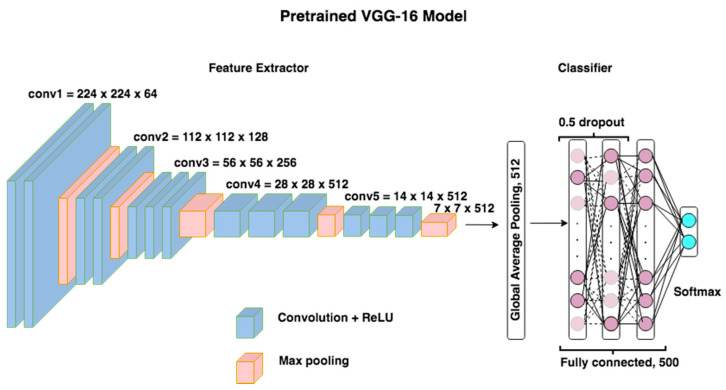
VGG-16 architecture.

Deep transfer learning was used by adopting a VGG-16 model, which is a standard convolutional neural network (CNN) pretrained on the huge ImageNet dataset [46], hence its weights have already been optimized on a different task. In this work, the pretrained VGG-16 model was trained to diagnose neonatal jaundice using our small dataset. Transfer learning can accelerate training time since weights are not randomly initialized, and therefore eliminates the need for large datasets. As shown in Figure 4, the VGG-16 model has two main parts, namely the feature extractor and the classifier.

#### 3.4.1. Feature Extraction

The feature extractor has an input layer of fixed size 224 × 224 RGB images, followed by thirteen convolutional layers, with a rectified linear unit activation function and five max-pool layers. The output of the feature extractor is deep-learned features of dimension 7 × 7 × 512.

#### 3.4.2. Classification

For the classifier part, the last fully connected layer of VGG-16 was removed, and the last maximum pooling layer in the feature extractor was connected to a global average pooling to convert the image features from a 7 × 7 × 512 vector to a 1 × 1 × 512 vector. Then, three dense layers with two dropout layers with 0.5 probability were added to avoid overfitting. Lastly, a Softmax function was used in the final layer.

### 3.5. Traditional Machine Learning

In this work, four well-known machine learning models were used, namely MLP, SVM, DT, and RF. Below, a brief explanation of the feature extraction and classification models is provided.

#### 3.5.1. Feature Extraction

Features were extracted from the segmentations of the neonate’s sclera and skin. Inspired by previous studies, such as [9,10,14], we extracted color features, RGB color space, then colormap transferred them to other color spaces such as YCbCr, Lab color space, and HSV color space. Then, the mean for color channel for each region was calculated, which resulted in 12 forehead skin features, 12 left eye features, and 12 right eye features, giving a total of 36 features.

#### 3.5.2. Classification

MLP is the classical type of neural networks with feed-forward neurons. Each neuron is a perceptron, which can take any number of inputs and produce a binary output. MLP consists of multiple fully connected layers, including input, output, and one or more hidden layers.

SVM is a robust well-known supervised learning model. The main goal of SVM is to find the optimal hyperplane that separates n-dimensional data into two classes. The optimal hyperplane is the one that maximizes the margin between the two classes of data. The margin represents the distance between the closest data points from each class to the hyperplane, which are called support vectors. When data is nonlinearly separable, SVM uses a kernel function to map the data into a higher dimension space, where it becomes linearly separable. The main parameters of SVM are C, the kernel function, and Gamma. The parameter C is used for regularization, while the kernel function determines the shape of the hyperplane, such as linear, RBF, and polynomial kernel. The Gamma hyper-parameter is set only with a Gaussian RBF kernel.

DT is a tree-structured learning algorithm which consists of two types of nodes, test/attribute nodes and class nodes. The former are internal nodes in the tree with two or more branches representing answers to the test, while the latter are leaf nodes. The root node is the most significant attribute based on a splitting metric such as information gain and GainRatio. Each test node partitions the data instances into two or more partitions according to the outcome of the test. This process is repeated until all instances in a partition belong to the same class. There are multiple DT algorithms such as ID3, C4.5, C5.0, and CART. In this work, we used an optimized version of CART implemented in Scikit-learn.

RF is an ensemble learning method that is used for both classification and regression. RF builds multiple DTs using bagging, i.e., bootstrap aggregation, where each DT is trained on a random subset of the data (bootstrap samples). The final output of the model is the aggregation of the DTs outputs using majority voting for classification or average for regression. Advantages of RF model is that it is diverse, stable, and immune to curse of dimensionality.

### 3.6. Evaluation

For evaluation, five-fold cross validation was used to train and test both transfer and traditional machine learning models. In addition, the positive class in the extracted structured dataset was oversampled using the Synthetic Minority Oversampling Technique (SMOTE) for the traditional machine learning models, while data augmentation was applied on the original image dataset for the deep transfer learning model in order to obtain balanced data. The performance of the models was evaluated using accuracy, precision, recall, F1 score, and the AUC score. Further, the k-fold cross-validated paired *t*-test [47] was applied in order to assess the statistical significance between two models A and B according to Equation (2) below.

(2) $$t=\frac{\bar{p}\;\sqrt{k}}{\sqrt{\frac{\sum_{i=1}^{k}{\left(p^{i}-\bar{p}\right)}^{2}}{k-1}}}$$

where *k* is the number of folds, $p^{i}$ is the difference between the model performances in the ith iteration $p^{i}=p_{A}^{i}-p_{B}^{i}$, and $\bar{p}$ computes the average difference between the model performances $\bar{p}=\frac{1}{k}\;\overset{k}{\underset{i=1}{\sum }}p^{i}$.

## 4. Results and Discussion

In this section, we present the experimental results for traditional and transfer machine learning models with respect to several performance metrics. The results presented with three types of features including skin, eye, and fusion of features. Each reported result is the average of five-fold cross validation. The parameters of the methods were instantiated based on the empirical experiments and by following the recommendations from the literature. We show parameter values used for the models in Table 3. *t*-tests were used to analyze and compare the performances of different combinations of features and classifiers.

The performance of the transfer learning model and traditional models, namely MLP, SVM, DT, and RF, is presented in Table 4 and Table 5, respectively, with respect to accuracy, precision, recall, F1 score, and AUC. In medical diagnosis problems, the main goal is to minimize false negative error, which is measured using recall. The first set of results for the transfer learning model are shown in Table 4. Interestingly, and in contrast to previous studies such as [17], the transfer learning model achieved the best performance with skin features rather than eye features. *t*-test showed that the performance of the model with skin features had significantly improved over eye features at *p* < 0.05 with respect to accuracy, recall, F1 score, and AUC (*p* = 0.04 for all performance measures), while no significant performance improvement was observed with respect to precision. When comparing the performance of the model with skin features and fused features, there were significant differences with respect to recall and AUC with *p* = 0.04, and no significant differences in performance with respect to accuracy, precision, and F1 score.

Our findings suggest that skin features are preferable with transfer learning since they improve the diagnosis performance of the model significantly with respect to most measures. These findings are in contrast to a widely perceived sense that eye features are better than skin features for jaundice diagnosis, such as in [17]. However, conclusions of previous studies were based on using statistical methods or traditional machine learning methods, rather than deep transfer learning. In this study, it was found that the set of the best features varied between traditional and transfer learning models. Consequently, conclusions made for traditional machine learning methods cannot be generalized for transfer learning models. Since the transfer learning model with skin features either exceeded or performed comparably to the model with fused features depending on the considered performance metric, it can be implied that fusing eye features with skin features for jaundice diagnosis using transfer learning did not contribute to improving performance, and hence can be disregarded.

The results of traditional learning models presented in Table 5 reveal several observations. First, we can see that, on average, the best diagnosis was achieved using the fused features. Overall, the recall of the models improved significantly with the fused features compared to that with the skin features at *p* < 0.05 (*p* = 0.0004). However, no significant difference in recall was achieved compared to that of eye features with *p* = 0.47. Similar performance trends were observed with respect to accuracy, precision, F1 score, and AUC.

Taken together, our results suggest that traditional machine learning models trained on eye features performed significantly better than when trained on skin features, which mirrors findings of previous studies [17]. Further, the results show that traditional machine learning models with eye features had comparable performance to those with fused features. This indicates that images of neonate skin had no significant contribution to improving diagnosis of neonatal jaundice when fused to eye images to train traditional machine learning models. Hence, when eye images are available, skin images can be overlooked as a source of data for diagnosing jaundice.

Table 4 also shows that MLP with eye features had the best jaundice diagnosis performance, followed by RF, SVM, and, lastly, DT. However, the *t*-test found no significant difference in recall performance between the former and RF and SVM at *p* < 0.05, with *p* = 0.34 and *p* = 0.2, respectively, while the diagnosis recall dropped significantly with DT with *p* = 0.02. Further statistical tests revealed that the same performance trends for the models were observed with respect to accuracy, precision, F1 score, and AUC. As for the fused features, it can be seen in Table 4 that MLP outperformed all other models, followed by SVM, RF, and, lastly, DT. However, statistical tests showed no significant performance difference between the models with respect to all performance measures.

These results imply that, among traditional machine learning models, MLP, SVM, and RF were the best jaundice diagnostic models. Further, they showed that although fusing skin features with eye features does not improve performance, it can make choosing a model for jaundice diagnosis a less important factor. The reason is that they improved the performance of the least performing model, i.e., DT, to make it perform comparably to other good models.

On comparing the best transfer learning performance, i.e., with skin features in Table 4, with the traditional machine learning model of best performance, namely MLP, SVM, and RF with eye features in Table 5, we can see that the former outperformed the latter with respect to all performance measures. However, *t*-test results showed that no significant difference was achieved between the performance of transfer learning and MLP with respect to all metrics, while a significant improvement was observed for transfer learning over SVM and DT with respect to accuracy with *p* = 0.02 and *p* = 0.04, respectively. This shows that using the right features for traditional learning models can make them compete with deep transfer model in some domains. However, as stated previously, the set of best features may vary between traditional and transfer learning models.

## 5. Conclusions

The goal of this work was to investigate the effectiveness of transfer learning in diagnosing neonatal jaundice using different types of features, namely skin, eye, and fusion of skin and eyes features. Moreover, the work aimed to compare transfer learning with traditional machine learning models, including multi-layer perceptron (MLP), support vector machine (SVM), decision tree (DT), and random forest (RF), when trained on the previously mentioned features. Our results showed that the transfer learning model performed the best with skin features, while traditional machine learning models achieved the best performance with eye features. For the traditional models, MLP, SVM, and RF models performed comparably with eye features and significantly better than the DT model. However, when using the fused features, all four models had similar performance. Further, the transfer learning model with skin features performed comparably to the MLP model with eye features. This showed that using the right features for traditional learning models could make them compete with a deep transfer model in some domains. Nonetheless, the right set of features may vary between traditional and transfer learning models.

## Figures and Tables

**Table 1:** Comparison of related works with their reported results.

Ref.	Feature Extraction	Method	Dataset	Result
[7]	Face skin (mean, standard deviation, skewness, kurtosis, energy, entropy)	K-Nearest Neighbours (KNN)	120 random images from Google infant monitoring	Accuracy = 90–96%
[8]	Forehead skin (RGB)	Linear regression model	64 images at Aalborg University Hospital in Denmark	Green sensitivity = 100%, specificity = 62% Blue sensitivity = 90%, specificity = 60%
[9]	Sternum and forehead skin (YCbCr and lab color spaces)	Ensemble of five regression algorithms (KNN, Least angle regression (LARS), LARS-Lasso Elastic Net, Support vector regression (SVR), Random forest (RF))	100 images collected from University of Washington Medical Center (UWMC) and the Roosevelt Pediatric Care Center	A linear correlation of 0.84 with TSB, with a mean error of 2.0 mg/dL
[10]	Forehead and sternum skin (Lab color spaces)	Matching	Standard set of serum bilirubin coloration on detection strips	Correlation = 0.93
[11]	Forehead skin (RGB and Hue, Saturation, Intensity (HIS) values)	Regression	113 images at Hafez and Shoushtari hospitals in Shiraz, Iran using a Samsung phone	Sensitivity = 68% Specificity = 92.3%
[12]	Sternum skin (YCbCr and lab color spaces)	Regression	530 images of different races in US including African American, Hispanic, and Asian American	Sensitivity = 84.6% Specificity = 75.1%
[13]	Sternum and abdomen skin (Hue and Saturation values)	Regression	35 images in Chennai, India	Sternum correlation = 0.6 Abdomen correlation = 0.55
[14]	Abdomen skin (YCbCr, RGB, and lab color spaces)	KNN SVR	80 image from Fırat University Faculty of Medicine, Neonatal Department in Turkey	KNN accuracy = 85% SVR accuracy = 75%
[15]	Soles, palm, forehead, and arm skin (RGB + diffuse reflectance spectra)	SVM	20 images of Mexican infants	Sensitivity = 71.8% Specificity = 78.8%
[16]	Face, arms, feet and middle body skin (RGB)	Linear regression	196 images at Firat university, Faculty of Medicine using an Android mobile phone or tablet	Accuracy = 92.5%
[17]	Eye (RGB)	Linear regression	110 images at University College London Hospital captured using a Nikon D3200 camera	Correlation = 0.75
[18]	Eye (sclera blue pixels)	Random forest regression	70 images of adults eyes at University of Washington using an iPhone SE	Sensitivity = 89.7% Specificity = 96.8%
[19]	Eye (RGB)	Regression	86 images at the UCH Neonatal Unit in London using a Nikon Dh3200 camera	Correlation = 0.71
[20]	Eye	Diazo method with dichloroaniline (DCA)	100 images at King Khalid Hospital at Al-Majma’ah, Saudi Arabia and Alpine Hospital, Gurgaon, India using a Samsung 10	Sensitivity = 92.0% specificity = 75.6%
[21]	Eye (PCA to extract L, a, and b values per CIE lab color)	Artificial neuro-fuzzy inference system (ANFIS)	420 images of adults’ eyes captured in fixed conditions using a 3CDD digital camera in aphotic housing made up of acrylic sheet	Accuracy = 90%
[22]	Eye (RGB)	Jaundice Eye Color Index Scleral-Conjunctival Bilirubin ((JECI-SCB) model and SCB_xy_ model	51 images from the UCL Hospital using an LG Nexus 5X smartphone	Correlation = 0.75
[38]	Bilirubin sample strips (homomorphic filter and blue color intensity)	Correlation between actual and predicted bilirubin level	8 images of bilirubin sample strips	Correlation coefficient increased from magnitude 0.5261 to magnitude 0.6974 after filtering

**Table 2:** Dataset characteristics.

Characteristic	Value
Dataset size (images)	68
Gender (images)	
Male	44
Female	24
Gestation age (weeks)	
Max.	42
Avg.	38
Min.	35
Age (days)	
Max.	5
Avg.	1
Min.	1
TCB level (Mmol/M)	
Max.	280
Avg.	135
Min.	0
Weight (kg)	
Max.	4
Avg.	3
Min.	2
Class (images)	
Healthy	44
Jaundiced	24

**Table 3:** Classification models parameter values.

Classification Model	Parameters Values
MLP	Loss = binary_crossentropy
	optimizer = Adam
	epochs = 50
	batch_size = 32
	layers = 2
	Hidden layers = 200
	Relu
	softmax
	Dropout = 0.5
SVM	Kernel = RBF
	C = 1000
	Gamma = 0.7
DT	Criterion = gini
	Splitter = best
	Max_depth = None
	min_samples_split = 2
	min_samples_leaf = 1
	min_weight_fraction_leaf = 0.0
	max_features = None
	random_state = None
	max_leaf_nodes = None
	min_impurity_decrease = 0.0
	min_impurity_split = 0
	class_weight = none
	ccp_alpha = 0.0
RF	n_estimators = 100
	criterion = gini
	max_depth = none
	min_samples_split = 2
	min_samples_leaf = 1
	min_weight_fraction_leaf = 0.0
	max_features = “auto”
	max_leaf_nodes = None
	min_impurity_decrease = 0.0
	min_impurity_split = None
	bootstrap = True
	oob_score = False
	n_jobs = None
	random_state = None
	verbose = 0
	warm_start = False
	class_weight = None
	ccp_alpha = 0.0
	max_samples = None
CNN	batch_size = 100
	epochs = 500
	momentum = 0.8
	SGD Optimizer

**Table 4:** Performance evaluation of the transfer learning model with different types of features.

Features	Accuracy	Precision	Recall	F1 Score	AUC
Skin	86.83%	84.49%	81.05%	82.12%	81.05%
Eye	79.03%	75.28%	69.67%	70.73%	69.67%
Fusion	79.95%	79.76%	71.25%	72.12%	71.25%

**Table 5:** Performance evaluation of traditional machine learning models with different types of features.

Features	Classifier	Accuracy	Precision	Recall	F1 Score	AUC
Skin	MLP	66.02%	66.26%	65.64%	64.47%	65.64%
	SVM	65.95%	69.42%	65.95%	64.60%	67.50%
	DT	62.35%	61.35%	61.18%	61.40%	60.89%
	RF	64.77%	72.54%	64.77%	61.50%	60.04%
	Avg.	64.77%	67.39%	64.39%	62.99%	63.52%
Eye	MLP	79.61%	80.62%	79.04%	78.84%	79.04%
	SVM	74.97%	75.97%	74.97%	74.70%	75.96%
	DT	62.35%	64.37%	62.22%	59.70%	60.25%
	RF	77.19%	77.58%	77.19%	77.10%	81.06%
	Avg.	73.53%	74.64%	73.36%	72.59%	74.08%
Fusion	MLP	77.62%	78.66%	77.62%	77.71%	77.41%
	SVM	76.41%	76.44%	76.41%	75.80%	82.01%
	DT	67.19%	70.65%	67.19%	69.8%	70.17%
	RF	72.75%	73.89%	72.75%	72.10%	78.86%
	Avg.	73.49%	74.91%	73.49%	73.85%	77.11%

## Data Availability

The data presented in this study are available on request from the corresponding author. The data are not publicly available due to the patients’ privacy.

## References

[1] Pagliari C., Sloan D., Gregor P., Sullivan F., Detmer D., Kahan J.P., Oortwijn W., MacGillivray S. (2005). What is eHealth (4): A scoping exercise to map the field. J. Med. Internet Res..

[2] Hsu C.-M., Hsu C.-C., Hsu Z.-M., Shih F.-Y., Chang M.-L., Chen T.-H. (2021). Colorectal Polyp Image Detection and Classification through Grayscale Images and Deep Learning. Sensors.

[3] Khasawneh N., Fraiwan M., Fraiwan L., Khassawneh B., Ibnian A. (2021). Detection of COVID-19 from Chest X-ray Images Using Deep Convolutional Neural Networks. Sensors.

[4] Umair M., Khan M.S., Ahmed F., Baothman F., Alqahtani F., Alian M., Ahmad J. (2021). Detection of COVID-19 Using Transfer Learning and Grad-CAM Visualization on Indigenously Collected X-ray Dataset. Sensors.

[5] Ahmedt-Aristizabal D., Armin M.A., Denman S., Fookes C., Petersson L. (2021). Graph-Based Deep Learning for Medical Diagnosis and Analysis: Past, Present and Future. Sensors.

[6] Maisels M.J. (2006). Neonatal jaundice. Pediatr. Rev..

[7] Mansor M.N., Hariharan M., Basah S.N., Yaacob S. (2013). New newborn jaundice monitoring scheme based on combination of pre-processing and color detection method. Neurocomputing.

[8] Munkholm S.B., Krøgholt T., Ebbesen F., Szecsi P.B., Kristensen S.R. (2018). The smartphone camera as a potential method for transcutaneous bilirubin measurement. PLoS ONE.

[9] De Greef L., Goel M., Seo M.J., Larson E.C., Stout J.W., Taylor J.A., Patel S.N. Bilicam: Using mobile phones to monitor newborn jaundice. Proceedings of the 2014 ACM International Joint Conference on Pervasive and Ubiquitous Computing.

[10] Saini N., Kumar A., Khera P. (2016). Non-Invasive Bilirubin Detection Technique for Jaundice Prediction Using Smartphones. Int. J. Comput. Sci. Inf. Secur..

[11] Padidar P., Shaker M., Amoozgar H., Khorraminejad-Shirazi M., Hemmati F., Najib K.S., Pourarian S. (2019). Detection of neonatal jaundice by using an android OS-based smartphone application. Iran. J. Pediatr..

[12] Taylor J.A., Stout J.W., de Greef L., Goel M., Patel S., Chung E.K., Koduri A., McMahon S., Dickerson J., Simpson E.A. (2017). Use of a smartphone app to assess neonatal jaundice. Pediatrics.

[13] Swarna S., Pasupathy S., Chinnasami B. (2018). The smart phone study: Assessing the reliability and accuracy of neonatal jaundice measurement using smart phone application. Int. J. Contemp. Pediatr..

[14] Aydın M., Hardalaç F., Ural B., Karap S. (2016). Neonatal Jaundice Detection System. J. Med. Syst..

[15] Castro-Ramos J., Toxqui-Quitl C., Manriquez F.V., Orozco-Guillen E., Padilla-Vivanco A., Sánchez-Escobar J.J. (2014). Detecting jaundice by using digital image processing. Three-Dimensional and Multidimensional Microscopy: Image Acquisition and Processing XXI.

[16] HARDALAÇ F., Aydin M., Kutbay U.Ğ., Ayturan K., AKYEL A., Çağlar A., HAi B., Mert F. (2021). Classification of neonatal jaundice in mobile application with noninvasive image processing methods. Turk. J. Electr. Eng. Comput. Sci..

[17] Leung T.S., Kapur K., Guilliam A., Okell J., Lim B., MacDonald L.W., Meek J. (2015). Screening neonatal jaundice based on the sclera color of the eye using digital photography. Biomed. Opt. Express.

[18] Mariakakis A., Banks M.A., Phillipi L., Yu L., Taylor J., Patel S.N. (2017). Biliscreen: Smartphone-based scleral jaundice monitoring for liver and pancreatic disorders. Proc. ACM Interact. Mob. Wearable Ubiquitous Technol..

[19] Outlaw F., Meek J., MacDonald L.W., Leung T.S. Screening for Neonatal Jaundice with a Smartphone. Proceedings of the 2017 International Conference on Digital Health.

[20] Rizvi M.R., Alaskar F.M., Albaradie R.S., Rizvi N.F., Al-Abdulwahab K. (2019). A Novel Non-invasive Technique of Measuring Bilirubin Levels Using BiliCapture. Oman Med. J..

[21] Laddi A., Kumar S., Sharma S., Kumar A. (2013). Non-invasive jaundice detection using machine vision. IETE J. Res..

[22] Outlaw F., Nixon M., Odeyemi O., MacDonald L.W., Meek J., Leung T.S. (2020). Smartphone screening for neonatal jaundice via ambient-subtracted sclera chromaticity. PLoS ONE.

[23] Parinyanut P., Bandisak T., Chiengkriwate P., Tanthanuch S., Sangkhathat S. (2016). Digital camera image analysis of faeces in detection of cholestatic jaundice in infants. African J. Paediatr. Surg..

[24] Ho E.Y.W., Lee S.Y.R., Chow C.B., Chung J.W.Y. (2006). BiliCheck transcutaneous bilirubinometer: A screening tool for neonatal jaundice in the Chinese population. Hong Kong Med. J..

[25] Boo N., Ishak S. (2007). Prediction of severe hyperbilirubinaemia using the Bilicheck transcutaneous bilirubinometer. J. Paediatr. Child Health.

[26] Hemmati F., Rad N.A.K. (2013). The value of Bilicheck^®^ as a screening tool for neonatal jaundice in the South of Iran. Iran. J. Med. Sci..

[27] Engle W.D., Jackson G.L., Stehel E.K., Sendelbach D.M., Manning M.D. (2005). Evaluation of a transcutaneous jaundice meter following hospital discharge in term and near-term neonates. J. Perinatol..

[28] Gomez M., Bielza C., Fernandez del Pozo J.A., Rios-Insua S. (2007). A graphical decision-theoretic model for neonatal jaundice. Med. Decis. Mak..

[29] American Academy of Pediatrics (2004). Subcommittee on Hyperbilirubinemia, Management of hyperbilirubinemia in the newborn infant 35 or more weeks of gestation. Pediatrics.

[30] Moyer V.A., Ahn C., Sneed S. (2000). Accuracy of clinical judgment in neonatal jaundice. Arch. Pediatr. Adolesc. Med..

[31] De Luca D., Zecca E., Zuppa A.A., Romagnoli C. (2008). The joint use of human and electronic eye: Visual assessment of jaundice and transcutaneous bilirubinometry. Turk. J. Pediatr..

[32] Riskin A., Abend-Weinger M., Bader D. (2003). How accurate are neonatologists in identifying clinical jaundice in newborns?. Clin. Pediatr..

[33] Gupta A., Kumar A., Khera P. Jaundice prediction through non-invasive techniques: Issues and challenges. Proceedings of the 2015 Annual IEEE India Conference (INDICON).

[34] Chou J.H. (2020). Predictive Models for Neonatal Follow-Up Serum Bilirubin: Model Development and Validation. JMIR Med. Inform..

[35] Azar A.T., Inbarani H.H., Kumar S.U., Own H.S. (2016). Hybrid system based on bijective soft and neural network for Egyptian neonatal jaundice diagnosis. Int. J. Intell. Eng. Inform..

[36] Ferreira D., Oliveira A., Freitas A. (2012). Applying data mining techniques to improve diagnosis in neonatal jaundice. BMC Med. Inform. Decis. Mak..

[37] Sajana B., Chandana T. (2018). A compration of unsupervised learning techniques in jaundice diagnosis. Int. J. Pure Appl. Math..

[38] Singla R., Singh S. A framework for detection of jaundice in new born babies using homomorphic filtering based image processing. Proceedings of the 2016 International Conference on Inventive Computation Technologies (ICICT).

[39] Hsu W.Y., Cheng H.C. (2021). A Fast and Effective System for Detection of Neonatal Jaundice with a Dynamic Threshold White Balance Algorithm. Healthcare.

[40] Robertson A., Kazmierczak S., Vos P. (2002). Improved transcutaneous bilirubinometry: Comparison of SpectR x BiliCheck and Minolta jaundice meter JM-102 for estimating total serum bilirubin in a normal newborn population. J. Perinatol..

[41] Szabo P., Wolf M., Bucher H.U., Fauchere J.-C., Haensse D., Arlettaz R. (2004). Detection of hyperbilirubinaemia in jaundiced full-term neonates by eye or by bilirubinometer?. Eur. J. Pediatr..

[42] Johnson S.M., Vasu V., Marseille C., Hill C., Janvier L., Toussaint P., Battersby C. (2020). Validation of transcutaneous bilirubinometry during phototherapy for detection and monitoring of neonatal jaundice in a low-income setting. Pediatr. Int. Child Health.

[43] Gasparini F., Schettini R. (2004). Color balancing of digital photos using simple image statistics. Pattern Recognit..

[44] Sharma S., Shanmugasundaram K., Ramasamy S.K. FAREC—CNN based efficient face recognition technique using Dlib. Proceedings of the 2016 International Conference on Advanced Communication Control and Computing Technologies (ICACCCT).

[45] Amos B., Ludwiczuk B., Satyanarayanan M. (2016). Openface: A general-purpose face recognition library with mobile applications. CMU Sch. Comput. Sci..

[46] Krizhevsky A., Sutskever I., Hinton G.E. (2017). Imagenet classification with deep convolutional neural networks. Commun. ACM.

[47] Dietterich T.G. (1998). Approximate statistical tests for comparing supervised classification learning algorithms. Neural Comput..

